# Renal denervation in hypertensive patients not on blood pressure lowering drugs

**DOI:** 10.1007/s00392-016-0984-y

**Published:** 2016-04-22

**Authors:** Rosa L. De Jager, Margreet F. Sanders, Michiel L. Bots, Melvin D. Lobo, Sebastian Ewen, Martine M. A. Beeftink, Michael Böhm, Joost Daemen, Oliver Dörr, Dagmara Hering, Felix Mahfoud, Holger Nef, Christian Ott, Manish Saxena, Roland E. Schmieder, Markus P. Schlaich, Wilko Spiering, Pim. A. L. Tonino, Willemien L. Verloop, Eva E. Vink, Evert-Jan Vonken, Michiel Voskuil, Stephen G. Worthley, Peter J. Blankestijn

**Affiliations:** 1 Department of Nephrology and Hypertension, University Medical Center Utrecht, Room F03.220, PO Box 85500, 3508 GA Utrecht, The Netherlands; 2 The Julius Center for Health Sciences and Primary Care, University Medical Center Utrecht, Utrecht, The Netherlands; 3 William Harvey Research Institute, Barts NIHR Cardiovascular Biomedical Research Unit, Queen Mary University of London, London, UK; 4 Klinik für Innere Medizin III, Kardiologie, Angiologie und Internistische Intensivmedizin, Universitätsklinikum des Saarlandes, Homburg/Saar, Germany; 5 Department of Cardiology, University Medical Center Utrecht, Utrecht, The Netherlands; 6 Department of Interventional Cardiology, Thoraxcenter, Erasmus Medical Center, Rotterdam, The Netherlands; 7 Department of Cardiology, University of Giessen, Giessen, Germany; 8 School of Medicine and Pharmacology-Royal Perth Hospital Unit, University of Western Australia, Perth, Australia; 9 Baker IDI Heart and Diabetes Institute, Melbourne, Australia; 10 Department of Nephrology and Hypertension, University Hospital of Erlangen, Erlangen, Germany; 11 Department of Vascular Medicine, University Medical Center Utrecht, Utrecht, The Netherlands; 12 Department of Cardiology, Heartcenter Catharina Hospital, Eindhoven, The Netherlands; 13 Department of Radiology, University Medical Center Utrecht, Utrecht, The Netherlands; 14 Cardiovascular Research Centre, University of Adelaide, Royal Adelaide Hospital, Adelaide, Australia

**Keywords:** Blood pressure reduction, Hypertension, Renal denervation, Sympathetic activity, Drug naive, Medication adherence

## Abstract

**Introduction:**

Studies on the blood pressure lowering effect of renal denervation (RDN) in resistant hypertensive patients have produced conflicting results. Change in medication usage during the studies may be responsible for this inconsistency. To eliminate the effect of medication usage on blood pressure we focused on unmedicated hypertensive patients who underwent RDN.

**Methods and results:**

Our study reports on a cohort of patients, who were not on blood pressure lowering drugs at baseline and during follow-up, from eight tertiary centers. Data of patients were used when they were treated with RDN and had a baseline office systolic blood pressure (SBP) ≥140 mmHg and/or 24-h ambulatory SBP ≥130 mmHg. Our primary outcome was defined as change in office and 24-h SBP at 12 months after RDN, compared to baseline. Fifty-three patients were included. There were three different reasons for not using blood pressure lowering drugs: (1) documented intolerance or allergic reaction (57 %); (2) temporary cessation of medication for study purposes (28 %); and (3) reluctance to take antihypertensive drugs (15 %). Mean change in 24-h SBP was −5.7 mmHg [95 % confidence interval (CI) −11.0 to −0.4; *p* = 0.04]. Mean change in office SBP was −13.1 mmHg (95 % CI −20.4 to −5.7; *p* = 0.001). No changes were observed in other variables, such as eGFR, body–mass-index and urinary sodium excretion.

**Conclusion:**

This explorative study in hypertensive patients, who are not on blood pressure lowering drugs, suggests that at least in some patients RDN lowers blood pressure.

**Electronic supplementary material:**

The online version of this article (doi:10.1007/s00392-016-0984-y) contains supplementary material, which is available to authorized users.

## Introduction

Sympathetic overactivity and kidney injury are major contributors in sustaining high blood pressure (BP) levels [1]. Percutaneous renal denervation (RDN) of the sympathetic nerves surrounding the renal arteries has been introduced as a therapy for (resistant) hypertension [2, 3]. Several studies have shown a reduction in ambulatory systolic blood pressure (SBP) ranging from 5 to 10 mmHg at 6- to 12-month follow-up after RDN [2, 4–6]. In the Symplicity HTN-3 trial, no difference in BP change between RDN-treated patients and the sham-treated control group was reported [7]. This has greatly fueled the discussion on the role of RDN as an antihypertensive treatment. Technical and procedural insufficiency may have hampered the proof of an antihypertensive effect of RDN [8]. In addition, it has been argued that the effects in earlier studies could be attributed to regression to the mean, improvement in lifestyle factors and, in particular, to a change in medication use [9–11]. In the Symplicity HTN-3 study, substantial differences in baseline anti-hypertensive medications and a striking 40 % change in prescribed anti-hypertensives in both control and RDN-treated groups during the study has seriously limited evaluation of the true effect of RDN [7]. Furthermore it is now well recognized that drug adherence in patients with hypertension is highly variable which further complicates assessment of anti-hypertensive effects of drugs or device therapy [12–14]. Recent RDN trials have attempted to overcome this problem by witnessed medication intake or by applying adherence questionnaires [6, 7, 15]. In these randomized controlled trials, the effect of RDN on 24-h SBP ranged from no change to a reduction of 6 mmHg, with comparable medication adherence in RDN treated patients and the control group. Hypertensive patients on no medication seem to be an ideal population to quantify the effect of RDN on BP. Furthermore, patients with intolerance of anti-hypertensive medication pose a major challenge to clinicians and novel approaches are needed to improve their BP control given their high cardiovascular risk [16]. This study reports on a collaborative initiative of eight centers active in device based therapy for hypertension. We present the results of RDN in hypertensive patients who used no blood pressure lowering drugs for their BP before RDN and during follow-up.

## Methods

### Design and study population

The study was designed to evaluate a cohort of patients that underwent RDN and who were either without blood pressure lowering drugs at baseline and follow-up, or, whose medication was withdrawn according to protocol. Our primary outcome was defined as change in office and 24-h SBP at 12 months after RDN, compared to baseline. Eight international centers (seven in Europe and one in Australia) participated in this initiative (Table 4, Supplemental Digital Content, which represents the participating centers). These centers delivered patient records that met the following inclusion criteria: the patient was ≥18-year-old, treated with catheter-based RDN and had a baseline office SBP ≥140 mmHg and/or 24-h SBP ≥130 mmHg. Patients were excluded if they were using medication for their hypertension or when no BP data were available at baseline or during follow-up visits. Local medical ethics committees approved the primary study in which the patient originally participated, in accordance with the Declaration of Helsinki.

### Blood pressure assessments

Twenty-four-hour BP and office BP measurements were collected at baseline and at 6 months and/or 12 months post RDN. Twenty-four-hour BP was calculated as the mean of the readings at least every 30 min at daytime and every hour at nighttime. Office BP was calculated as the mean of three measurements obtained with a noninvasive automatic blood pressure measuring device with at least 5 min resting between each BP reading. All BP measurements were performed in accordance with the European guidelines and with recommended devices [17, 18]. In the absence of a control group, we compared our results with the possible BP lowering effect of simply taking part in a study. To assess this potential placebo effect, we selected studies from a recently published systematic review by Patel and co-workers (Fig. 2, Supplemental Digital Content, represents a forest plot of the selected studies) [19].

### Other assessments

We collected physical (e.g., height, weight) and biochemical parameters (e.g., urinary sodium excretion) to explore lifestyle and other potentially relevant factors at baseline and follow-up. We report on body mass index, kidney function and 24-h urinary sodium excretion. Serum creatinine was determined as standard care at each study site (Jaffé or Enzymatic method). The estimated glomerular filtration rate (eGFR) was calculated using the Chronic Kidney Disease Epidemiology Collaboration (CKD-EPI) or Modification of Diet in Renal Diseases (MDRD) equation [20, 21]. Measurements were standardized by converting the creatinine measurements with the Jaffé method to the Enzymatic method and the eGFR with MDRD to the CKD-EPI estimation.

### RDN procedure

Study sites selected patients for RDN according to their own study protocol (Table 4, Supplemental Digital Content, which represents the participating centers). Percutaneous radiofrequency ablation was performed with Symplicity^TM^ catheter (Medtronic Inc., Santa Rosa, California) or EnligHTN^TM^ Ablation catheter (St Jude Medical, St Paul, MN, USA). Ultrasound RDN was performed with the use of PARADISE^TM^ technology (ReCor Medical, Ronkonkoma, NY, USA). The treating physician decided which renal arteries to treat, which device to use and how many ablations could be performed.

### Statistical analysis

Results are presented as the mean difference between baseline and 12 months with corresponding standard error and 95 % CI interval, unless otherwise stated. When the 95 % CI does not contain the zero value, the difference is considered statistically significant. Our primary outcome was change in BP 12 months after RDN. For missing data, we used the 6-month BP data carried forward. The rationale for this approach was to increase the number of individuals with an outcome variable. This was considered to be reasonable based on previous reports showing that over time the magnitude of the RDN effect does not seem to attenuate between 6 and 12 months, if anything an increase in RDN effect is expected [5, 22]. To study the mean changes in BP we used paired analyses. To study change in BP and change in biological variables after RDN, we applied a linear regression model. Also, a linear regression model was applied to explore which baseline factors were related to the blood pressure change. Univariable models were the main approach due to the small sample size. To explore the data further, we applied a one-way ANOVA model to determine whether the reason for not using blood pressure lowering drugs resulted in different BP changes. In the present study we aimed to collect results of as many individuals as possible, who underwent RDN and were not using blood pressure lowering drugs. Therefore, no sample size estimation was done upfront. All analyses were performed using the IBM SPSS Statistics for Windows, Version 21.0 (IBM Corp., Armonk, NY, USA).

## Results

### Baseline characteristics

Fifty-three records of patients, who complied with our inclusion criteria, were included. There were three different reasons for not using BP lowering drugs: (1) documented intolerance or allergic reaction (57 %); (2) temporary cessation of medication for study purposes (followed by immediate resumption of drug treatment after study visits), using a highly standardized stepwise program (28 %); and (3) reluctance to take antihypertensive drugs (15 %) [23, 24]. Four patients for whom the reason was unknown were included in the first group. All patients underwent RDN between May 2011 and August 2014 in different study settings (Table 4, Supplemental Digital Content). Baseline characteristics are summarized in Table 1. Mean baseline 24-h BP was 160 ± 17/94 ± 11 mmHg and mean office BP was 180 ± 24/101 ± 14 mmHg. Mean baseline eGFR estimated by CKD-EPI was 85 ± 18 ml/min/1.73 m^2^. Three patients (6 %) had moderately reduced kidney function (eGFR <60 ml/min/1.73 m^2^). Forty-two patients were treated with the Symplicity catheter, ten with the EnligHTN catheter and one was treated with ultrasound RDN. Baseline characteristics of the three groups of patients, according to the reason for not using blood pressure lowering drugs, are shown in Table 5 (Supplemental Digital Content).

**Table 1:** Baseline characteristics of the study population

	All patients (*n* = 53)
Age (years)^a^	62 (35–80)
Gender (male)^b^	24 (45.3)
Caucasian^b^	53 (100)
Body mass index	28.4 (±4.9)
Comorbidity	
Dyslipidemia^b^	36 %
Diabetes Mellitus type 2^b^	11 %
Cardiovascular diseases^b^	15 %
Cerebrovascular diseases^b^	6 %
Current smoking^b^	4 (8)
Nr. of antihypertensive drugs^a^	0 (0–0)
Reason for no medication use	
Intolerance, unknown^b^	30 (57)
Study purposes^b^	15 (28)
Never prescribed^b^	8 (15)
Office blood pressure	
Systolic (mmHg)	180 (±24)
Diastolic (mmHg)	101 (±14)
Heart rate (bpm)	72 (±10)
Ambulatory blood pressure	
24-h systolic (mmHg)	160 (±17)
24-h diastolic (mmHg)	94 (±11)
24-h heart rate (bpm)	72 (±9)
eGFR, CKD epi (mL/min/1.73 m^2^)	85 (±18)
Presence of accessory renal arteries^b^	13 (25)
Not all renal arteries treated^b^	7 (15)
Device used	
Symplicity^b^	42 (79)
EnligHTN^b^	10 (19)
PARADISE^b^	1 (2)
Nr. of ablations^a^	13 (2–25)

Data are expressed as mean ± SD, unless stated otherwise

Body mass index is the weight in kilograms divided by the square of the height in meters

*Bpm* beats per minute, *eGFR* estimated glomerular filtration rate

^a^Data are mean (range)

^b^Data are *n* (%) or percentage

### Change in blood pressure

Twenty-four-hour BP and office BP data were available in 43 and 47 patients, respectively (6-month office and 24-h BP data were carried forward for 7 and 14 patients, respectively). In the whole group, 24-h SBP and diastolic BP (DBP) reduced after RDN as compared to baseline by −5.7 mmHg [95 % confidence interval (CI), −11.0 to −0.4; *p* = 0.04] and −4.0 mmHg (95 % CI −6.6 to −1.4; *p* = 0.003), respectively. Office SBP and DBP decreased significantly after RDN by −13.1 mmHg (95 % CI −20.4 to −5.7; *p* = 0.001) and −4.4 mmHg (95 % CI −7.8 to −1.1; *p* = 0.01), respectively (Table 2). There were no statistically significant differences in BP change between the three groups (*p* = 0.45 and *p* = 0.93 for 24-h SBP and office SBP, respectively) (Table 6, Supplemental Digital Content). BP changes at 6 and 12 months are separately presented in Table 7 (Supplemental Digital Content). Based on a systematic review, a selective pooling of previous studies was performed to assess the effect of participating in a trial on BP levels. Mean change in office SBP in the placebo controlled group was −4.0 mmHg (95 % CI −7.5 to −0.4) and the change in 24-h SBP −0.9 mmHg (95 % CI −2.1 to 0.2) (Fig. 2, Supplemental Digital Content, which represents a forest plot of the selected studies).

**Table 2:** Change in blood pressure and other relevant parameters after RDN

	*N*	Mean change compared to baseline (95 % CI)
Ambulatory blood pressure		
24-h systolic (mmHg)	43	−5.7 (−11.0 to −0.4)
24-h diastolic (mmHg)	43	−4.0 (−6.6 to −1.4)
24-h heart rate (bpm)	35	−1.1 (−3.8 to 1.7)
Day-time systolic (mmHg)	39	−8.2 (−13.4 to −3.0)
Day-time diastolic (mmHg)	39	−4.9 (−7.9 to −2.5)
Nighttime systolic (mmHg)	38	−6.3 (−14.1 to 1.4)
Nighttime diastolic (mmHg)	38	−4.8 (−9.9 to 0.4)
Office blood pressure		
Systolic (mmHg)	47	−13.1 (−20.4 to −5.7)
Diastolic (mmHg)	47	−4.4 (−7.8 to −1.1)
Heart rate (bpm)	25	−2.6 (−6.7 to 1.5)
Body mass index (kg/m^2^)	25	0.5 (−0.9 to 1.9)
eGFR, CKD epi (mL/min/1.73 m^2^)	48	0.4 (−1.9 to 2.8)
Urinary sodium excretion (mmol/24 h)	16	−23.3 (−89.3 to 42.7)

*N* represents the number of patients with information on the variable of interest at baseline and at follow-up

Body mass index is the weight in kilograms divided by the square of the height in meters

*Bpm* beats per minute, *eGFR* estimated glomerular filtration rate

### Anatomic and procedural determinants

**Fig. 1:**
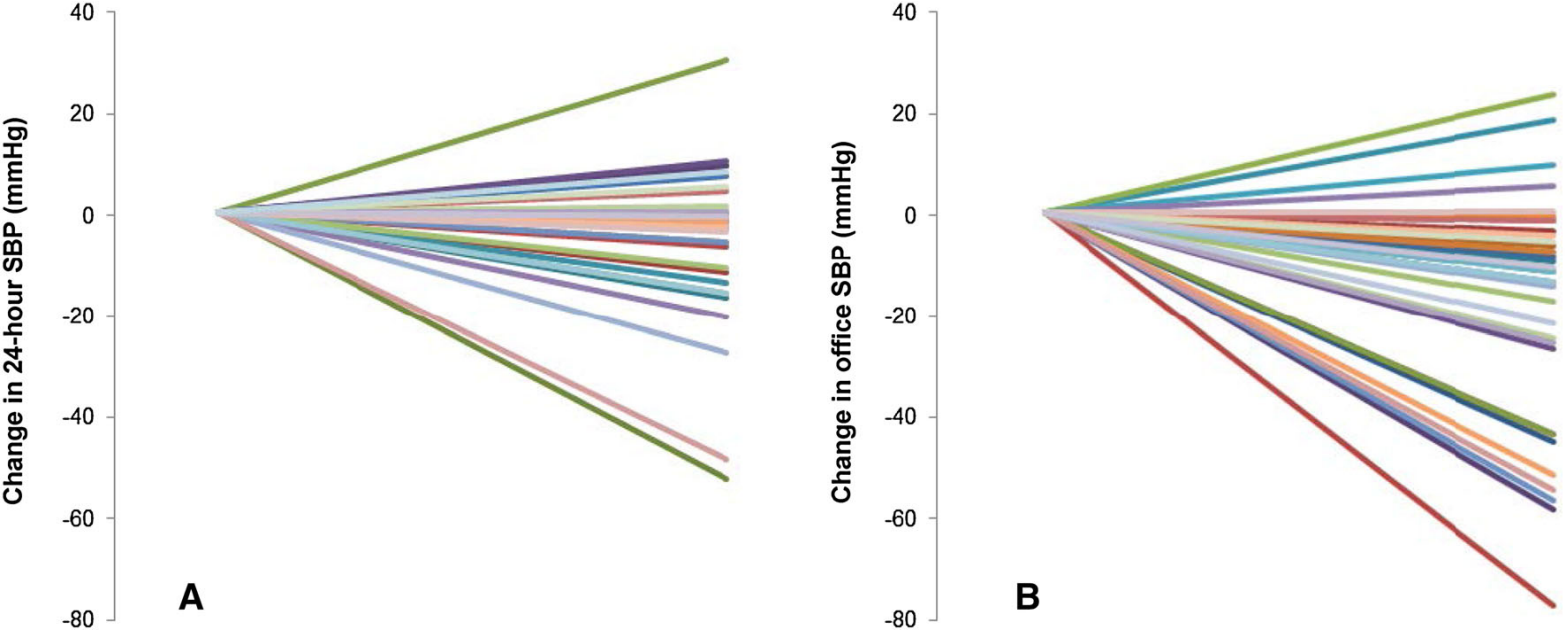
Individual changes in blood pressure after RDN, in patients with solitary renal arteries **a**, *n* = 35 and **b**, *n* = 34. *SBP* systolic blood pressure

Renal artery anatomy was established in 50 patients. Thirty-seven patients had a solitary artery on both sides, 13 patients had accessory renal arteries on one or both sides, of which three patients had more than one. Patients with solitary renal arteries were all treated in both renal arteries. Of the patients having accessory renal arteries, seven patients could not be treated in all renal arteries. In Fig. 1, the individual changes in BP are presented for the patients with solitary renal arteries. Mean change in 24-h SBP is −5.4 mmHg (95 % CI −10.7 to −0.11) and mean change in office SBP is −18.5 mmHg (95 % CI −26.7 to −10.4). Individual changes of the patients with accessory renal arteries are shown in Fig. 3 (Supplemental Digital Content). Change in 24-h SBP and office SBP did not differ between groups based on the device (Symplicity and EnligHTN) used for RDN (*p* = 0.56; *p* = 0.87, respectively). There was no relation between the number of ablations and the change in 24-h SBP and office SBP (*p* = 0.97; *p* = 0.71, respectively). Data are not shown in this article.

### Explorative analyses into determinants of response to RDN

Univariable analysis showed no significant relation between baseline 24-h SBP and change in 24-h SBP after RDN [mean change in 24-h SBP is −0.22 mmHg (95 % CI −0.53 to 0.083; *p* = 0.15) for every mmHg increase in baseline 24-h SBP]. There was a significant relation between baseline office SBP and change in SBP after RDN (mean change in office SBP is −0.36 mmHg (95 % CI −0.64 to −0.089; *p* = 0.011) for every mmHg increase in baseline office SBP).We observed a relation between percentage dipping at baseline and change in SBP after RDN [mean change in 24-h SBP is 0.76 mmHg (95 % CI 0.18 to 1.35; *p* = 0.01) and for office SBP 0.82 mmHg (95 % CI 0.013 to 1.62; *p* = 0.047] for every percentage increase in dipping (Fig. 4, Supplemental Digital Content, which represents the relation between these variables). This demonstrates that patients with more nocturnal dipping have less reduction in blood pressure after RDN. Furthermore, nighttime BP was positively related to change in SBP after RDN [mean change in 24-h SBP is −0.43 mmHg (95 % CI −0.70 to −0.16; *p* = 0.002) and for office SBP −0.35 mmHg (95 % CI −0.74 to −0.054; *p* = 0.088) for every percentage increase in nighttime BP]. All univariable analyses are presented in Table 3. With regard to lifestyle and other biological factors, we observed no changes in BMI, eGFR and urinary sodium excretion after RDN (Table 2).

**Table 3:** Univariable analyses of change in 24-h systolic blood pressure

	*N*	B (95 % CI)
Age	43	0.19 (−0.39 to 0.76)
Gender, female	43	1.58^a^ (−9.23 to 12.38)
Body mass index	40	0.52 (−0.53 to 1.58)
eGFR, CDK epi (mL/min/1.73 m^2^)	40	−0.13 (−0.45 to 0.18)
Urine sodium mmol/24 h	24	0.01 (−0.05 to 0.07)
Baseline 24-h SBP (mmHg)	43	−0.22 (−0.53 to 0.08)
Baseline percentage dipping	38	0.76 (0.18 to 1.35)
Baseline nighttime SBP (mmHg)	38	−0.43 (−0.70 to −0.16)
Nr. of ablations	40	−0.03 (−1.75 to 1.69)

Univariable analyses of the relation between baseline and/or procedural characteristics and the change in 24-h systolic blood pressure after RDN in all patients

Body mass index is the weight in kilograms divided by the square of the height in meters

*SBP* systolic blood pressure

*N* represents the number of patients with information on both the change in 24-h systolic blood pressure and the variable of interest

*B* the regression coefficient, reflects the mean change in 24-h systolic blood pressure by one unit increase in determinant

^a^B reflects the mean change in 24-h systolic blood pressure if this characteristic is applied

## Discussion

To the best of our knowledge, this is the first report on the BP lowering effect of RDN in hypertensive patients who were not using blood pressure lowering drugs at baseline and during follow-up. Ambulatory and office BP were significantly reduced after RDN, in this patient group with considerable heterogeneity. So far, the effect of RDN has been investigated when added to medical therapy in patients with so called resistant hypertension. Resistant hypertension is defined as an office SBP ≥140 mmHg, despite the use of at least three BP lowering drugs [17]. A major difficulty in such studies is that use of prescribed medication is highly variable and, importantly, may change over time. In the present study, this poorly controllable, but important effect modifier, has been eliminated by selecting patients not on antihypertensive drugs, allowing an estimation of the net effect of RDN. The magnitude of the RDN effect seen in our study is comparable to what has been documented in the DENERHTN study, in which the BP lowering efficacy of RDN plus standardized antihypertensive treatment was compared with standardized antihypertensive treatment alone in patients with resistant hypertension. In DENERHTN specific efforts were undertaken to maximize medication adherence [6]. When looking at 6-months results, they noted a change in 24-h BP of −5.9/−3.1 mmHg which is not very different from the −5.0/−2.0 mmHg we found in our study. In addition, we found a further decline to −7.0/−4.0 mmHg 12 months after RDN. As mentioned above, we observed considerable heterogeneity of BP response to RDN. This variability was also noted in previous studies [6, 22]. Procedure and patient related factors could play a role. The majority of the renal denervation procedures were done with Medtronic’s Simplicity device. It is now increasingly clear that procedural factors such as completeness of circumferential coverage, depth and location of ablations may result in a variable and unpredictable degree of nerve destruction and as result a variable effect on BP [25, 26]. In this small study sample, we found no relation between the number of ablations and BP effect and no difference in effect between the two devices. Explorative analyses were performed on patient related factors that may affect the degree of effect. As consistently reported earlier, we found that a higher baseline office SBP is associated with a larger BP reduction [22, 27, 28]. Interestingly, the BP lowering effect was larger in non-dipping patients. This finding is in line with the knowledge that reduced nocturnal dipping is a characteristic of an upregulated sympathetic nervous system [24]. Furthermore, a comparable relation between nighttime BP and reduction in BP was seen.

For this study, we collected records of patients previously treated with RDN, therefore a control group was lacking. This results in uncertainty whether the observed decline in BP after RDN may (partially) be due to other mechanisms, including lifestyle improvement, the effect of taking part in a trial and also the ‘regression to the mean’ phenomenon. Our data suggest no major changes in potentially relevant factors, such as BMI, eGFR and urinary sodium excretion. It is highly implausible that ‘regression to the mean’ can be responsible when observing sustained BP reductions 12 months post RDN. Furthermore, most patients already have a long history of hypertension. To overcome the limitation of having no control group, we assessed the BP lowering effect in the placebo arm of hypertension trials in patient populations not on antihypertensive drugs, based on a recently published systematic review. (Figure 2, Supplemental Digital Content) [19, 29–38]. The comparison of this estimated placebo effect with the present analysis suggests that the 24-h and office BP reduction after RDN (−5.7 and −13.0 mmHg systolic, respectively) is on average larger than could be expected from participating in a study per se (−0.9 and −4.0 mmHg change in SBP, respectively). Although, we believe this is the best available comparison, an important limitation is the heterogeneity of these studies and, on average, lower baseline BP compared with our study. Furthermore, the calculated study-/placebo effect was purely based on pharmacological interventions. The effect of a sham procedure might be different.

This study has some other limitations as well. Firstly, our study may consist of a highly selected population. However, when compared to earlier studies, our population did not differ in mean levels of predictors of response to RDN [6, 7, 15, 22, 39]. Therefore, our results unlikely reflect a biased estimate. Secondly, we did not measure drug metabolites to check whether patients were really not using blood pressure medication during the measurement. However, it seems unlikely that patients are using drugs without prescription.

## Conclusion

In conclusion, this explorative study suggests a beneficial effect of RDN on blood pressure in patients with hypertension, independent of medication change during the study. Furthermore, this supports the rationale to investigate the effects of RDN in a patient population not on blood pressure lowering drugs [40, 41].

## Electronic supplementary material

Below is the link to the electronic supplementary material.

Supplementary material 1 (DOCX 176 kb)
