# Supplementary material for: Renal denervation in hypertensive patients not on blood pressure lowering drugs
Source: Clin Res Cardiol. 2016 Apr 22;105:755–62. doi: 10.1007/s00392-016-0984-y (PMC4989018; doi:10.1007/s00392-016-0984-y)
Supplement: Supplementary file 1 — Supplementary material 1 (DOCX 176 kb) [file 392_2016_984_MOESM1_ESM.docx]

**ONLINE SUPPLEMENT**

**List of Supplemental Digital Content**

Supplemental Digital Content 1. Table 4 Overview of participating centers

Supplemental Digital Content 2. Table 5 Baseline characteristics by reason for not taking blood pressure lowering drugs

Supplemental Digital Content 3. Table 6 Blood pressure changes in strata of the reason for not taking blood pressure lowering drugs during the blood pressure measurements

Supplemental Digital Content 4. Table 7 Blood pressure at 6 and 12 months compared with baseline blood pressure

Supplemental Digital Content 5. **Fig. 2** Forest plot of studies examining the effect of being in a trial and receiving a placebo on the change in systolic blood pressure over time

Supplemental Digital Content 6. **Fig. 3** Individual changes in BP after RDN, in patients with accessory renal arteries (a, n=8 and b, n=13)

Supplemental Digital Content 7. **Fig. 4** The relation between percentage dipping at baseline and change in SBP after RDN

| Table 4 Overview of participating centers | | | |
| --- | --- | --- | --- |
| **Participating centers** | **Primary outcome** | **N** | **Pubmed ID** |
| University Medical Center Utrecht, the Netherlands  Registry  DREAMS | blood pressure  insulin sensitivity and blood pressure | 22  8 | 25326543  25646297 |
| Barts Health Trust and the William Harvey Research Institute London, UK | blood pressure | 6 | NA |
| Universitätsklinikum Erlangen, Germany | albuminuria | 3 | 24681017 |
| Universitätsklinikum Gießen, Germany | blood pressure | 3 | NA |
| Bakers IDI Heart and Diabetes Institute, Melbourne, Australia | blood pressure | 3 | NA |
| Universitätsklinikum des Saarlandes, Germany | blood pressure | 2 | NA |
| Erasmus Medical Center Rotterdam, the Netherlands | blood pressure | 1 | NA |
| Catharina Ziekenhuis Eindhoven, the Netherlands | blood pressure | 1 | NA |
| Global SYMPLICITY Registry | blood pressure | 4 | 25691618 |

RDN: renal denervation; N: number of patients without blood pressure lowering drugs; ID: identification number; NA: not applicable.

| Table 5 Baseline characteristics by reason for not taking blood pressure lowering drugs | | | |
| --- | --- | --- | --- |
|  | **Intolerance**  **(n=30)** | **Study purposes (n=15)** | **Never prescribed**  **(n=8)** |
| Age (years) | 61 (±12) | 59 (±9) | 58 (±10) |
| Gender, female ^ƚ^ | 20 (67) | 5 (33) | 4 (50) |
| Body-mass index (kg/m2) | 27.6 (±4.7) | 28.9 (±5.5) | 30.2 (±4.5) |
| Comorbidity |  |  |  |
| Dyslipidemia | 27% | 64% | 29% |
| Diabetes Mellitus type 2 | 3% | 27% | 13% |
| Cardiovascular diseases | 15% | 27% | 0% |
| Cerebrovascular diseases | 11% | 0% | 0% |
| Current smoking | 9% | 7% | 14% |
| Nr. of antihypertensive drugs^ϕ^ | 0 (0-0) | 0 (0-0) | 0 (0-0) |
| Office blood pressure |  |  |  |
| Systolic (mmHg) | 181 (±25) | 185 (±24) | 167 (±22) |
| Diastolic (mmHg) | 100 (±13) | 105 (±14) | 101 (±14) |
| Heart rate (bpm) | 72 (±11) | 72 (±8) | UN |
| Ambulatory blood pressure |  |  |  |
| 24-hour systolic (mmHg) | 159 (±14) | 170 (±17) | 145 (±18) |
| 24-hour diastolic (mmHg) | 94 (±10) | 98 (±13) | 88 (±13) |
| 24-hour heart rate (bpm) | 72 (±8) | 72 (±10) | 73 (±9) |
| eGFR, CDK epi (mL/min/1.73m2) | 85 (±16) | 81 (±20) | 90 (±17) |
| Presence of accessory renal arteries^ƚ^ | 7 (23) | 3 (20) | 3 (38) |
| Not all renal arteries treated ^ƚ^ | 3 (11) | 3 (20) | 1 (13) |
| Device used |  |  |  |
| Symplicity^ƚ^ | 21 (70) | 13 (87) | 8 (100) |
| EnligHTN^ƚ^ | 8 (27) | 2 (13) | 0 (0) |
| PARADISE^ƚ^ | 1 (3) | 0 (0) | 0 (0) |
| Nr. of ablations^ϕ^ | 13 (6-25) | 12 (10-16) | 13 (2-17) |

Data are expressed as mean ±SD, unless stated otherwise. Bpm, beats per minute; eGFR, estimated glomerular filtration rate; UN, unknown. Body-mass index is the weight in kilograms divided by the square of the height in meters.
ϕ Data are mean (range)
ƚ Data are n (%) or percentage

| Table 6 Blood pressure changes in strata of the reason for not taking blood pressure lowering drugs during the blood pressure measurements | | | |
| --- | --- | --- | --- |
|  | Intolerance | Study purpose | Never prescribed |
| Ambulatory blood pressure | N=22 | N=15 | N=6 |
| 24-hour systolic (mmHg) | -3.7 (-12.4 to 5.0) | -10.2 (-18.1 to -2.3) | -1.8 (-17.4 to 13.8) |
| 24-hour diastolic (mmHg) | -2.3 (-5.9 to 1.3) | -6.7 (-11.7 to -1.8) | -3.3 (-11.1 to 4.5) |
| 24-hour heart rate (bpm) | -1.8 (-6.4 to 2.9) | -0.15 (-2.8 to 2.5) | 0.0 (-25.4 to 25.4) |
| Daytime systolic (mmHg) | -7.1 (-16.0 to 1.8) | -10.3 (-18.9 to -1.8) | -6.5 (-20.0 to 7.0) |
| Daytime diastolic (mmHg) | -3.2 (-6.6 to 0.19) | -6.1 (-11.5 to -0.6) | -6.8 (-13.6 to -0.1) |
| Office blood pressure | N=29 | N=10 | N=8 |
| Systolic (mmHg) | -14.1 (-24.0 to -4.2) | -12.1 (-34.6 to 10.4) | -10.5 (-19.9 to -1.1) |
| Diastolic (mmHg) | -3.1 (-7.7 to 1.5) | -7.1 (-16.4 to 2.2) | -5.9 (-11.4 to -0.4) |
| Heart rate (bpm) | -3.2 (-7.7 to 1.2) | 2.0 (-19.5 to 23.5) | UN |

Data are expressed as mean change compared to baseline (95% CI). When all groups are compared, P=0.45 and P=0.93 for 24-hour systolic blood pressure and office systolic blood pressure, respectively.
N represents the number of patients with information on the variable of interest at baseline and at follow-up.
Bpm, beats per minute; UN, unknown.

| Table 7 Blood pressure at 6 and 12 months compared with baseline blood pressure | | | | | | | | | |
| --- | --- | --- | --- | --- | --- | --- | --- | --- | --- |
| Ambulatory blood pressure | N | Baseline | 6 months | P-value^*^ | Ambulatory blood pressure | N | Baseline | 12 months | P-value^*^ |
| 24-hour systolic (mmHg) | 19 | 159 (±15) | 154 (±22) | 0.314 | 24-hour systolic (mmHg) | 36 | 160 (±18) | 153 (±19) | 0.016 |
| 24-hour diastolic (mmHg) | 19 | 92 (±9) | 90 (±10) | 0.032 | 24-hour diastolic (mmHg) | 36 | 93 (±12) | 89 (±12) | 0.004 |
| 24-hour heart rate (bpm) | 17 | 73 (±9) | 71 (±10) | 0.283 | 24-hour heart rate (bpm) | 30 | 72 (±9) | 71 (±9) | 0.774 |
| Daytime systolic (mmHg) | 15 | 164 (±17) | 158 (±23) | 0.218 | Daytime systolic (mmHg) | 35 | 165 (±18) | 156 (±19) | 0.001 |
| Daytime diastolic (mmHg) | 15 | 95 (±9) | 92 (±11) | 0.032 | Daytime diastolic (mmHg) | 35 | 97 (±12) | 92 (±12) | 0.001 |
| Office blood pressure |  |  |  |  | Office blood pressure |  |  |  |  |
| Systolic  (mmHg) | 36 | 177 (±25) | 164 (±28) | 0.004 | Systolic  (mmHg) | 33 | 177 (±23) | 166 (±28) | 0.013 |
| Diastolic  (mmHg) | 36 | 98 (±13) | 94 (±14) | 0.036 | Diastolic  (mmHg) | 33 | 100 (±12) | 94 (±14) | 0.008 |
| Heart rate  (bpm) | 22 | 73 (±11) | 70 (±10) | 0.786 | Heart rate  (bpm) | 21 | 74 (±11) | 69 (±12) | 0.163 |

Data are expressed as mean ±SD. Bpm, beats per minute.

*P-value for the difference in mean blood pressure from baseline to 6 or 12 months follow-up.

**Figures supplementary data**


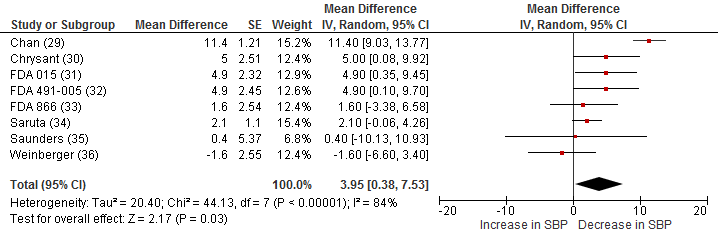
**A**

**
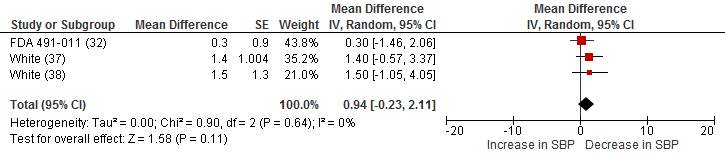
**

**B**

**Fig. 2** Forest plot of studies examining the effect of being in a trial and receiving a placebo on the change in systolic blood pressure (SBP) over time (A: office SBP ; B: 24-hour SBP). We selected from Patel et al. those that met the following criteria: no use of blood pressure lowering drugs in the control group and a follow-up ≥ 12 weeks. Results were obtained using a random effects model, weighted by using the inverse of standard error.[42] The mean differences of 4.0 mmHg and 0.9 mmHg reflect a reduction in SBP.


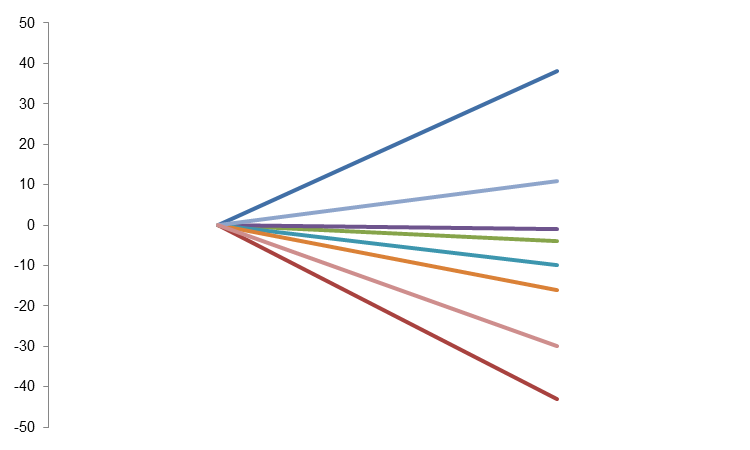

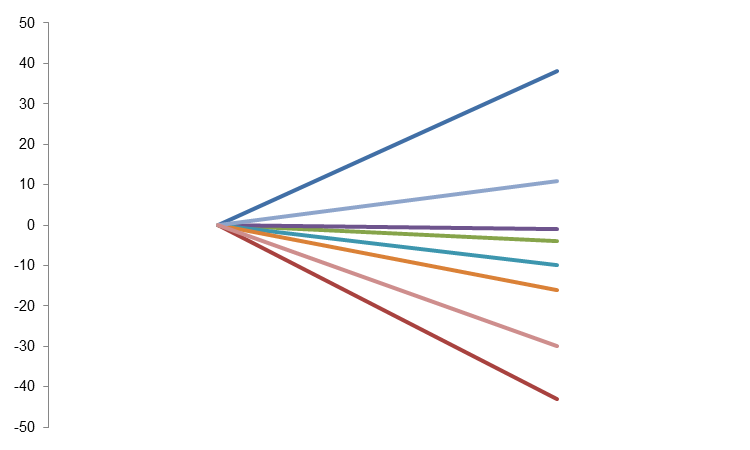

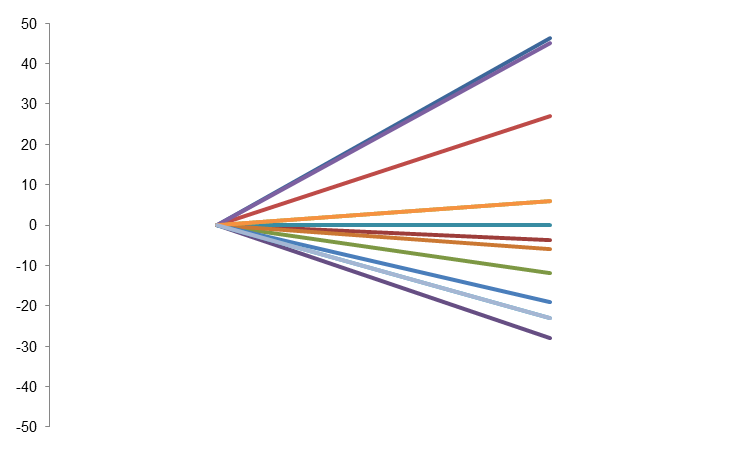

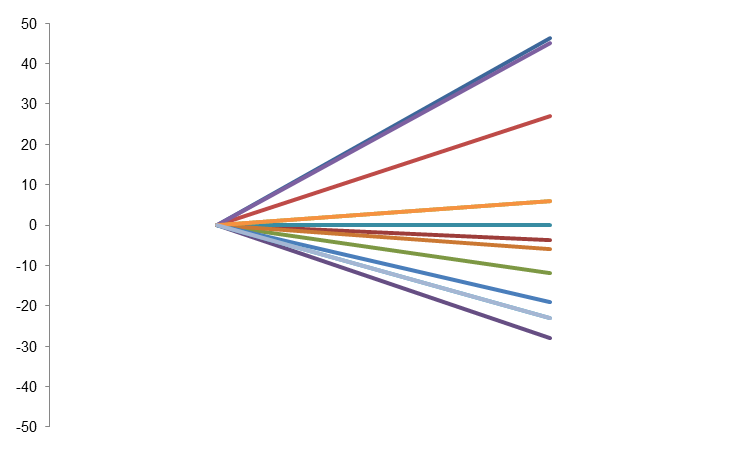


**A**

**B**

**Change in 24-hour SBP (mmHg)**

**Change in office SBP (mmHg)**

**Fig. 3** Individual changes in BP after RDN, in patients with accessory renal arteries (A, n=8 and B, n=13)
SBP, systolic blood pressure


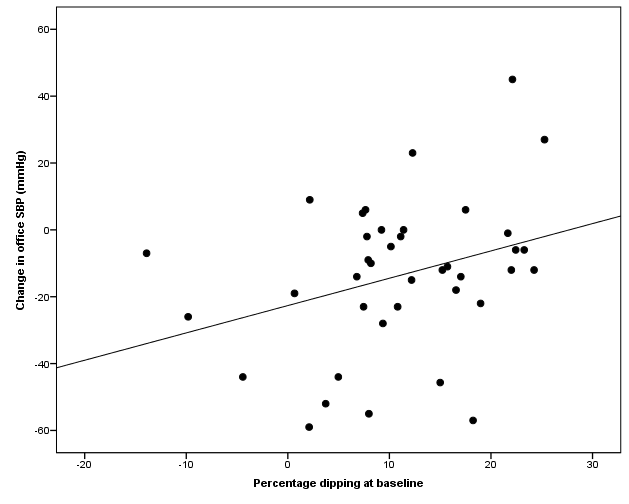

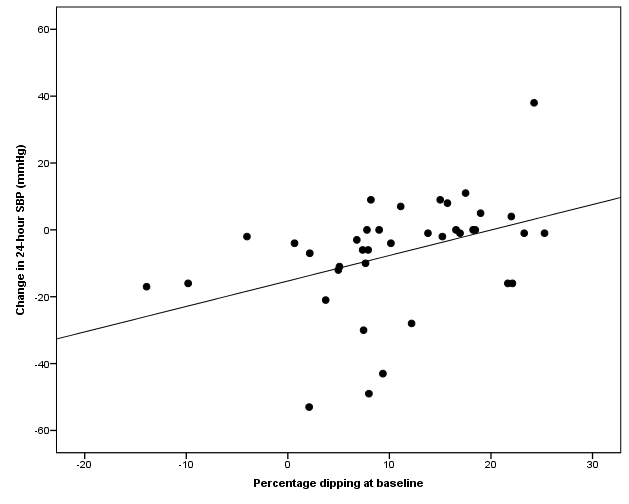


**A**

**B**

**Fig. 4** The relation between percentage dipping at baseline and change in SBP after RDN. For every percentage increase in dipping at baseline, the mean change in 24-hour SBP is 0.76 mmHg (A) and for office SBP 0.82 mmHg after RDN (B).This means that patients with more nocturnal dipping have less reduction in BP after RDN. SBP, systolic blood pressure
